# Comparative performance of large language models for patient-initiated ophthalmology consultations

**DOI:** 10.3389/fpubh.2025.1673045

**Published:** 2025-09-22

**Authors:** Mingxue Huang, Xiaoyan Wang, Shiqi Zhou, Xinyu Cui, Zilin Zhang, Yanwu Xu, Weihua Yang, Wei Chi

**Affiliations:** ^1^School of Nursing, Southwest Medical University, Luzhou, China; ^2^School of Future Technology, South China University of Technology, Guangzhou, China; ^3^Shenzhen Eye Hospital, Shenzhen Eye Medical Center, Southern Medical University, Shenzhen, China

**Keywords:** large language model, healthcare, consultation, ophthalmology, patient education

## Abstract

**Background:**

Large language models (LLMs) are increasingly accessed by lay users for medical advice. This study aims to conduct a comprehensive evaluation of the responses generated by five large language models.

**Methods:**

We identified 31 ophthalmology-related questions most frequently raised by patients during routine consultations and subsequently elicited responses from five large language models: ChatGPT-4o, DeepSeek-V3, Doubao, Wenxin Yiyan 4.0 Turbo, and Qwen. A five-point likert scale was employed to assess each model across five domains: accuracy, logical consistency, coherence, safety, and content accessibility. Additionally, textual characteristics, including character, word, and sentence counts, were quantitatively analyzed.

**Results:**

ChatGPT-4o and DeepSeek-V3 achieved the highest overall performance, with statistically superior accuracy and logical consistency (*p* < 0.05). Existing safety evaluations indicate that both Doubao and Wenxin Yiyan 4.0 Turbo exhibit significant security deficiencies. Conversely, Qwen generated significantly longer outputs, as evidenced by greater character, word, and sentence counts.

**Conclusion:**

ChatGPT-4o and DeepSeek-V3 demonstrated the highest overall performance and are best suited for laypersons seeking ophthalmic information. Doubao and Qwen, with their richer clinical terminology, better serve users with medical training, whereas Wenxin Yiyan 4.0 Turbo most effectively supports patients’ pre-procedural understanding of diagnostic procedures. Prospective randomized controlled trials are required to determine whether integrating the top-performing model into pre-consultation triage improves patient comprehension.

## 1 Introduction

Advances in deep learning have enabled large language models (LLMs) to achieve substantial breakthroughs in natural language processing, demonstrating broad utility across text generation, semantic comprehension, translation, and inferential reasoning (1, 2). Recently, generative artificial intelligence has exhibited considerable promise within the healthcare sector, particularly in standardized examination simulation and clinical documentation, thereby invigorating contemporary medical practice (3–5). Advanced LLMs, exemplified by ChatGPT and DeepSeek, are now systematically deployed across diverse medical specialties and have demonstrated early efficacy in disease recognition, diagnostic support, and evidence-based clinical decision-making (6–8). LLMs have demonstrated high diagnostic accuracy and decision-making efficacy in subspecialties such as neuro-ophthalmology, glaucoma, and thyroid eye disease, underscoring their substantial application potential (9–11). Furthermore, large language models such as Qwen, Doubao, and Wenxin Yiyan exhibit substantial translational promise across clinical and research workflows (12, 13). These systems enhance healthcare service efficiency, mitigate clinician workload, and foster patient health literacy and equitable access to care (14).

Nevertheless, the deployment of LLMs in clinical settings faces several challenges, among which ‘model hallucination’ is particularly pronounced (15, 16). Such models may produce outputs that are structurally coherent yet factually erroneous, a limitation arising from outdated training corpora and restricted access to contemporary medical guidelines, ultimately compromising the comprehensiveness and authority of their knowledge bases (17, 18). Although initiatives such as DeepSeek seek to mitigate the black-box problem through enhanced transparency and interpretability, the medical community retains circumspection regarding their reliability (19, 20). The growing utilization of LLMs for unsupervised health self-diagnosis may expose lay users to inaccurate or unsafe information, thereby amplifying potential harms (21, 22). Besides, ophthalmology necessitates exceptionally high diagnostic precision, as even marginal deviations can adversely affect patient prognosis (23). Therefore, comprehensive performance evaluations within ophthalmological contexts are urgently required prior to their widespread clinical adoption (24). Existing studies focus primarily on different versions of ChatGPT, leaving a scarcity of comparative analyses across models (25).

This study systematically evaluates five LLMs (ChatGPT-4o, DeepSeek-V3, Qwen, Doubao, and Wenxin Yiyan 4.0 Turbo) and focuses on their responses to ophthalmology-related questions from patients. Model outputs will be comprehensively assessed across five domains: accuracy, logical consistency, coherence, safety, and content accessibility. Additionally, quantitative text metrics (character, word, and sentence counts) will be extracted from Chinese-language outputs to elucidate their practical utility for patient education and clinical decision support.

## 2 Method

### 2.1 Ethical statement

This cross-sectional evaluation compared responses generated by five LLMs to 31 frequently encountered consultation questions in ophthalmology. The questions were derived from routine clinical inquiries collected by healthcare providers during patient encounters. Crucially, the study involved no patient-level data or personally identifiable information, thereby fully preserving individual anonymity and privacy.

### 2.2 Model selection

We purposefully selected five state-of-the-art LLMs: ChatGPT-4o, DeepSeek-V3, Qwen, Doubao, and Wenxin Yiyan 4.0 Turbo. Selection criteria encompassed recent benchmark performance, public accessibility, the developers’ institutional credibility, and demonstrated suitability for medical question-answering.

#### 2.2.1 ChatGPT-4o

ChatGPT-4o^1^ is OpenAI’s newest transformer-based large-scale language model. It leverages deep-learning techniques to deliver advanced generative and comprehension capabilities, and its multimodal architecture ensures robust performance across heterogeneous input modalities, encompassing text and images.

#### 2.2.2 DeepSeek-V3

DeepSeek-V3^2^ is engineered for high-performance information retrieval and open-domain question answering, integrating deep-learning and reinforcement-learning techniques to optimize retrieval efficiency and accuracy.

#### 2.2.3 Qwen

Qwen^3^ is a conversational LLM optimized for interactive question-answering, emphasizing user engagement and real-time feedback.

#### 2.2.4 Doubao

Doubao^4^ is specifically optimised for Chinese-language tasks, employing multi-layer attention mechanisms to capture nuanced semantics and cultural contexts.

#### 2.2.5 Wenxin Yiyan 4.0 Turbo

Wenxin Yiyan 4.0 Turbo^5^ is tailored for Chinese natural-language processing, exhibiting strong generative and semantic-understanding capabilities.

### 2.3 Study design

We conducted a cross-sectional benchmarking study evaluating how the five selected LLMs respond to 31 frequently asked consultation questions covering retinal diseases, macular degeneration, glaucoma, dry eye and associated procedures. Questions were classified as definitional, causal, comparative, or procedural and reflect typical patient queries.

On 6 March 2025, two investigators jointly recorded the answer generated by each model in a single submission. Each question was submitted separately through the online platforms corresponding to the five models. No system prompts were provided, and responses were generated *de novo* from the query. Following response generation, the chat histories were manually reset to prevent carryover of context.

All outputs were independently verified by two researchers and transcribed into a Microsoft Excel spreadsheet. Character, word, and sentence counts were automatically derived using the online text analytics tool Xiezuocat.^6^

Two board-certified vitreoretinal attending physicians with equivalent seniority (each with ≥5 years of subspecialty experience) independently rated each response across five domains: accuracy, logical consistency, coherence, safety, and content accessibility, using a five-point Likert scale (1 = poor, 5 = excellent). Detailed scoring criteria and the full question list are provided in the Supplementary material. All interactions were conducted within a controlled online environment following standardized operating procedures to maximize reproducibility.

### 2.4 Data analysis

All analyses were conducted in SPSS software (version 27.0). Inter-rater consistency of total scores was assessed with the intraclass correlation coefficient (ICC). Normality was evaluated using the Shapiro–Wilk test. Normally distributed continuous variables were expressed as mean ± SD; non-normally distributed variables as median (IQR). Homogeneity of variances was evaluated using Levene’s test. Parametric comparisons among the five models employed one-way analysis of variance (ANOVA); non-parametric analyses utilized the Kruskal–Wallis *H* test. Where significant differences were detected, Bonferroni-corrected *post-hoc* pairwise comparisons were performed. *p* < 0.05 was deemed statistically significant.

## 3 Results

### 3.1 Comparative performance of five LLMs

**Figure 1:**
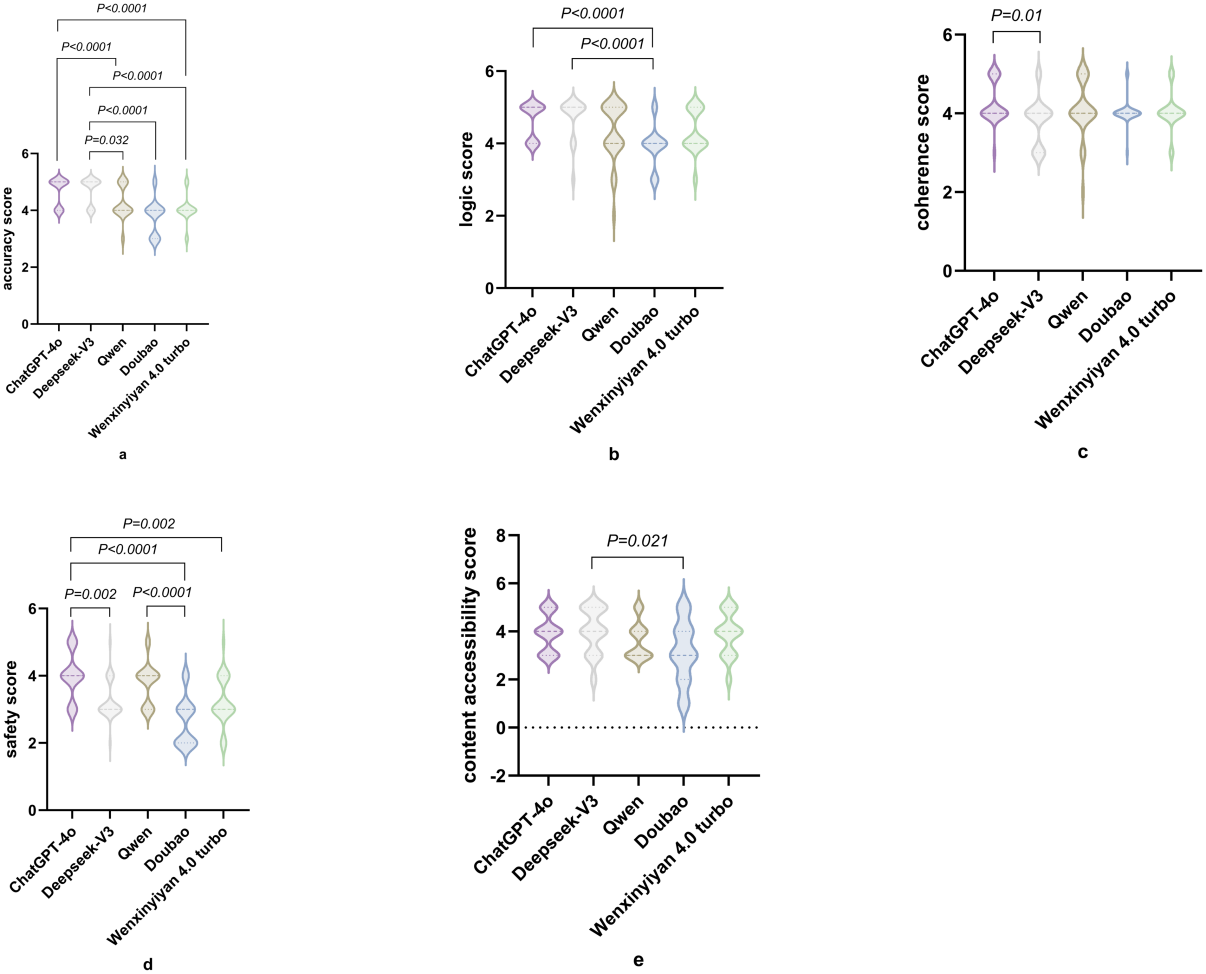
Pairwise comparisons between models. **(a)** Accuracy comparison; **(b)** Logic scores; **(c)** Coherence scores; **(d)** Safety scores; **(e)** Content accessibility scores.

The ICC between the two raters was 0.87. Table 1 summarizes the median scores of the five LLMs across five domains: accuracy, logical consistency, coherence, safety, and content accessibility. Accuracy: ChatGPT-4o and DeepSeek-V3 attained the maximum median score of 5.0, significantly surpassing the remaining models (*H* = 50.90, *p* < 0.05). Logical consistency: Likewise, ChatGPT-4o and DeepSeek-V3 achieved a median of 5.0, significantly exceeding the others (*H* = 29.82, *p* < 0.05). Coherence: Scores differed modestly; nevertheless, ChatGPT-4o and DeepSeek-V3 exhibited marginally higher stability (*H* = 11.69, *P*<0.05). Safety: ChatGPT-4o scored highest (4.0), whereas Doubao and Wenxin Yiyan 4.0 Turbo recorded the lowest (3.0), with significant between-group differences (*H* = 52.30, *p* < 0.05). Content accessibility: ChatGPT, DeepSeek-V3 and Wenxin Yiyan 4.0 Turbo performed best (4.0), while Qwen and Doubao scored lower (3.0); these differences were statistically significant (*H* = 12.54, *p* < 0.05). Detailed differences are provided in Table 2 and Figure 1.

**Table 1:** Performance scores of five large language models across accuracy, logic, coherence, safety, and content accessibility.

Metric	Chat GPT-4o	DeepSeek-V3	Qwen	Doubao	Wenxin Yiyan 4.0 turbo	*H*-value	*p*-value
Accuracy	5.0 (4.0,5.0)	5.0 (4.0,5.0)	4.0 (4.0,5.0)	4.0 (3.0,4.0)	4.0 (4.0,4.0)	50.90	<0.05
Logic	5.0 (4.0,5.0)	5.0 (5.0,5.0)	4.0 (4.0,5.0)	4.0 (4.0,4.0)	4.0 (4.0,5.0)	29.82	<0.05
Coherence	4.0 (4.0,5.0)	4.0 (3.0,4.0)	4.0 (4.0,5.0)	4.0 (4.0,4.0)	4.0 (4.0,4.0)	11.69	<0.05
Safety	4.0 (4.0,4.0)	3.0 (3.0,4.0)	4.0 (3.0,4.0)	3.0 (2.0,3.0)	3.0 (3.0,4.0)	52.30	<0.05
Content accessibility	4.0 (3.0,5.0)	4.0 (3.0,5.0)	3.0 (3.0,4.0)	3.0 (2.0,4.0)	4.0 (3.0,5.0)	12.54	<0.05

**Table 2:** Pairwise comparisons between models.

Comparison	Accuracy	Logic	Coherence	Safety	Content accessibility	Characters	Words	Sentences
ChatGPT-4o vs. DeepSeek-V3	1.000	1.000	0.010*	0.002*	1.000	0.091	0.037*	1.000
ChatGPT-4.o vs. Qwen	0.063	1.000	1.000	1.000	1.000	0.002*	0.006*	0.312
ChatGPT-4o vs. Doubao	0.000*	0.000*	0.866	0.000*	0.072	1.000	1.000	0.000*
ChatGPT-4o vs. Wenxin Yiyan 4.0 turbo	0.000*	0.431	0.927	0.002*	1.000	1.000	1.000	1.000
DeepSeek-V3 vs. Qwen	0.032*	0.234	0.195	0.057	0.848	0.000*	0.000*	0.077
DeepSeek-V3 vs. Doubao	0.000*	0.000*	1.000	0.065	0.021*	0.083	0.017*	0.002*
DeepSeek-V3 vs. Wenxin Yiyan 4.0 turbo	0.000*	0.078	1.000	1.000	1.000	0.001*	0.000*	1.000
Qwen vs. Doubao	0.060	0.077	1.000	0.000*	1.000	0.003*	0.014*	0.000*
Qwen vs. Wenxin Yiyan 4.0 turbo	1.000	1.000	1.000	0.052	1.000	0.180	0.369	0.058
Doubao vs. Wenxin Yiyan 4.0 turbo	1.000	0.231	1.000	0.071	0.121	1.000	1.000	0.004*

* denotes statistical significance at the 0.05 level.

### 3.2 Output length characteristics

**Figure 2:**
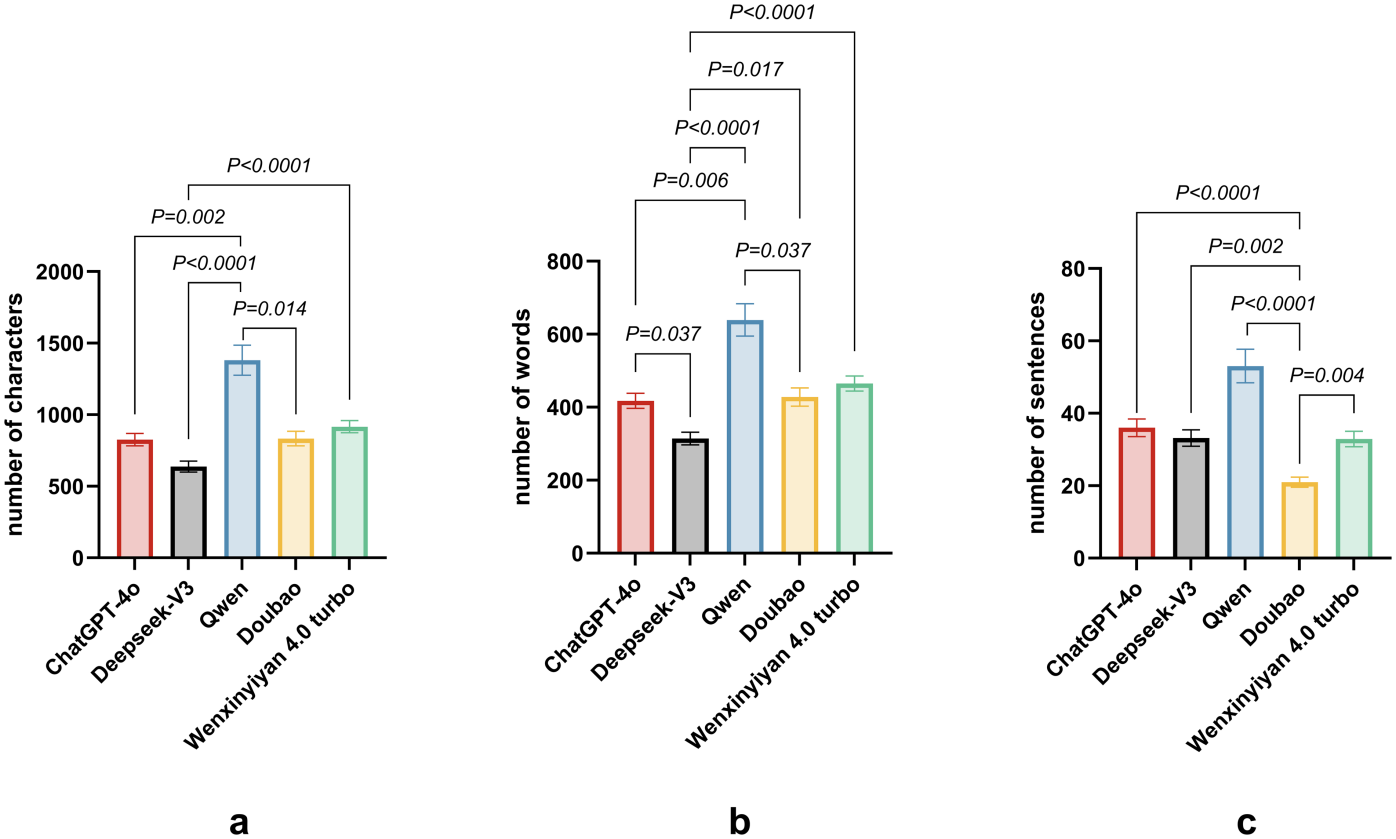
Response lengths of five large language models to 31 common consultation questions in ophthalmic practices. **(a)** Character count; **(b)** Word count; **(c)** Sentence count.

Table 3 and Figure 2 present descriptive statistics for character, word, and sentence counts. Qwen produced the longest responses (1,380.58), significantly exceeding ChatGPT-4o (826.48) and DeepSeek-V3 (636.90) (*p* < 0.05). Wenxin Yiyan 4.0 Turbo generated 916.45 words, approximating ChatGPT-4o. Similarly, Qwen yielded the highest token count (639.16), substantially surpassing DeepSeek-V3 (314.45) and ChatGPT-4o (417.55) (*p* < 0.05). Doubao and Wenxin Yiyan 4.0 Turbo produced fewer tokens (428.03 and 465.00, respectively). Qwen also generated the greatest number of sentences (53.06), significantly exceeding DeepSeek-V3 (33.16) and ChatGPT-4o (36.00) (*p* < 0.05). Conversely, Doubao and Wenxin Yiyan 4.0 Turbo produced the fewest sentences (20.97 and 32.90, respectively). Collectively, Qwen generated significantly more characters, words, and sentences than all other models (*p* < 0.05). Comprehensive pairwise comparisons are presented in Table 3 and Figure 2.

**Table 3:** Response lengths of five large language models to 31 ophthalmology related queries.

Metric	Chat GPT-4o	DeepSeek-V3	Qwen	Doubao	Wenxin Yiyan 4.0 turbo	*H*-value	*p*-value
Characters	826.48 ± 240.62	636.90 ± 213.66	1380.58 ± 584.93	833.29 ± 285.08	916.45 ± 237.45	41.94	<0.05
Words	417.55 ± 115.32	314.45 ± 96.95	639.16 ± 247.72	428.03 ± 140.17	465.00 ± 114.65	42.29	<0.05
Sentences	36.00 ± 13.63	33.16 ± 12.689	53.06 ± 25.77	20.97 ± 7.71	32.90± 11.80	41.52	<0.05

## 4 Discussion

As LLMs are increasingly adopted in ophthalmology, where diagnostic precision is paramount, their accuracy, safety, and clarity directly affect clinical decision support and patient education (26). Patients now commonly seek online health information and may obtain LLM-based advice without clinician oversight; therefore, these systems must meet rigorous quality standards before healthcare implementation.

Our findings demonstrate statistically significant inter-model heterogeneity, with ChatGPT-4o and DeepSeek-V3 achieving superior overall performance. Consistent with earlier reports (23, 27), ChatGPT-4o exhibits near-expert proficiency in ophthalmological question-answering, while DeepSeek-V3 matches ChatGPT-4o in accuracy; both significantly outperform the remaining three models. This superiority may be attributable to: (1) the increased complexity of open-ended questions relative to prior multiple-choice formats; (2) delayed updates in competing models; and (3) advanced algorithmic architectures and curated training corpora employed by ChatGPT-4o and DeepSeek-V3.

Coherence scores were comparable across models, yet ChatGPT-4o and Qwen exhibited marginally superior stability (28), suggesting that architectural heterogeneity influences medical reasoning construction. We additionally assessed the inclusion of disclaimers intended to mitigate medical and legal risk. ChatGPT-4o and Qwen frequently appended disclaimers (e.g., “seek prompt medical attention” or “consult a qualified clinician”), indicating stronger safety-control mechanisms than their counterparts.

When addressing different query types, all models provided comprehensive descriptions of disease-related content, particularly for definitional questions. For diagnostic tasks, DeepSeek-V3 and Wenxin Yiyan 4.0 Turbo supplied extensive clinical context and complication analyses, whereas ChatGPT-4o remained concise yet superficial (14). Previous studies have not reported that Doubao and Qwen display broader stylistic variation than other models (29), whereas ChatGPT-4o, although clear and concise, shows limited stylistic flexibility.

Upon addressing the query “How does diabetes induce retinal damage?,” ChatGPT-4o first defined diabetic retinopathy and summarized its pathophysiology, then listed preventive measures (glycemic control, annual retinal screening, optimization of lipids and blood pressure, smoking cessation, limited alcohol intake, and supplementation with lutein, vitamins C and E, and *ω*-3 fatty acids). DeepSeek-V3 more deeply into the underlying molecular mechanisms while simultaneously elaborating on disease progression and clinical manifestations. Qwen and Doubao concentrate on a hierarchical analysis of pathological mechanisms, whereas clinical management recommendations are comparatively sparse. Wenxin Yiyan 4.0 Turbo first described the disease, then detailed relevant examinations such as optical coherence tomography. The examples of this study indicate that ChatGPT-4o and DeepSeek-V3 are better suited for the general public seeking disease information, whereas Qwen, Wenxin Yiyan 4.0 Turbo, and Doubao employ more complex medical terminology that benefits clinical trainees but may hinder comprehension among non-specialists. Such complexity may impede information acquisition, emotional support, and interpersonal rapport among patients (30, 31).

Converging evidence from our multi-dimensional assessment described above suggests that the observed balance of accuracy, conciseness, and safety renders these models operationally viable for eye-care pathways.

Previous studies have demonstrated that ChatGPT demonstrates diagnostic accuracy comparable to, or even exceeding, that achieved by ophthalmology residents in distinguishing primary from secondary glaucoma (10). This study further demonstrated that ChatGPT-4o rapidly identified patients requiring immediate referral versus routine follow-up, consistent with earlier studies (32). Within hierarchical diagnostic and treatment settings, chatbots demonstrate a superior capacity to identify acute and severe conditions (33), substantially enhancing patient satisfaction and the overall care experience (34). In circumstances where a patient cannot attend a hospital or clinic in person, or requires expeditious triage to ascertain the urgency of professional medical attention, LLMs can be leveraged to provide case-specific recommendations (34).

The application of LLMs in ophthalmology is rapidly expanding across medical education, clinical support, research, and patient education (35). However, persistent challenges (inconsistent performance, algorithmic bias, hallucinations, data-privacy risks, and ethical dilemmas) remain (36). Patients with ophthalmic concerns should continue to consult certified eye-care professionals, ensuring adequate human oversight in clinical decision-making (26, 37). Future initiatives must prioritize iterative model refinement and interdisciplinary ethical governance to ensure responsible clinical deployment (24, 25). Empirical evidence confirms that well-crafted prompts enhance both output accuracy and contextual relevance (38–40), although prompt variation exerts limited influence on accuracy, it substantially modifies textual readability (41, 42). Consequently, readability remains pivotal for effective patient communication even when accuracy gains are marginal.

LLMs trained with domain-specific ophthalmological expertise outperform those trained on general corpora (43). Future validation pipelines for ophthalmology-focused LLMs should span multi-center, multi-tier institutions and establish an iterative cycle of fine-tuning, validation, and governance. Interdisciplinary experts in ophthalmology, law, and ethics will craft an adaptive governance framework, while curated multi-center datasets drive continuous model refinement. The integration of this model into medical education platforms can be used to generate immersive virtual patient cases that significantly bridge the gap between theory and clinical practice (44), while alleviating the healthcare burden in resource-limited regions (45, 46). We therefore recommend that the platform adopt a two-pronged strategy: first, encourage physicians to participate as cohesive teams to leverage peer-learning and collaborative mechanisms for enhancing overall service quality; second, embed robust privacy-preserving safeguards within personalized services so that patients can fully benefit from precision medicine without concerns about data security.

Our study has several limitations. First, each query was presented only once without priming or real-world outcome validation, potentially underestimating model capabilities. Second, analyses were restricted to Chinese-language responses, limiting generalizability. Third, we focused on the most common ophthalmic conditions, which may not fully capture the breadth of LLM functions. Future work should incorporate diverse, real-time datasets and develop validated tools for assessing linguistic complexity in Chinese LLMs to improve reliability, and should expand evaluation to additional models to clarify domain-specific strengths and limitations.

## 5 Conclusion

This study systematically evaluated five mainstream LLMs on ophthalmology question-answering tasks, revealing inter-model differences in accuracy, logical consistency, coherence, safety, and content accessibility. ChatGPT-4o and DeepSeek-V3 consistently outperformed the others, particularly in accuracy and logical consistency. Qwen produced the longest and most lexically rich outputs. Qwen, Wenxin Yiyan 4.0 Turbo, and Doubao employed complex medical terminology that may hinder comprehension among non-specialists. Continued technological advances and mitigation of current limitations will substantially enhance the clinical utility of LLMs.

## Data Availability

The original contributions presented in the study are included in the article/Supplementary material, further inquiries can be directed to the corresponding authors.

## Footnotes

^1^ https://openai.com/index/hello-gpt-4o/

^2^ https://chat.deepseek.com/

^3^ https://qwen.org/chat

^4^ https://www.doubao.com/chat/

^5^ https://yiyan.baidu.com/

^6^ https://xiezuocat.com/
