# Supplementary material for: Comparative performance of large language models for patient-initiated ophthalmology consultations
Source: Front Public Health. 2025 Sep 22;13:1673045. doi: 10.3389/fpubh.2025.1673045 (PMC12497754; doi:10.3389/fpubh.2025.1673045)
Supplement: Supplementary file 1 [file Table_1.DOCX]

**Problems**

**Section I. Definition Questions**

1.What is retinal detachment?

2.What is dry age-related macular degeneration?

3.What is neovascular (wet) age-related macular degeneration?

4.What is diabetic retinopathy?

5.What are optic neuropathies?

6.What is fundus hemorrhage?

7.What is astigmatism?

**Section II. Causal Relationship Questions**

1.Does wearing corrective lenses exacerbate the progression of myopia?

2.Which adverse habits in daily life and study may contribute to the risk of retinal detachment?

3.How does chronic hyperglycemia affect ocular health?

4.How does cigarette smoking increase the risk of developing age-related macular degeneration?

5.If there is a family history of glaucoma, does it guarantee that one will develop glaucoma?

6.Why do patients with glaucoma develop visual field defects?

7.What are the mechanisms by which diabetes leads to retinal damage?

**Section III. Comparison Questions**

1.What are the differences between glaucoma and cataract?

2.How do dry and neovascular (wet) age-related macular degeneration differ?

3.What is the distinction between retinal detachment and retinal tear?

4.What are the differences between acute angle-closure glaucoma and chronic open-angle glaucoma?

5.What is the difference between presbyopia and myopia?

6.What is the relationship between elevated intraocular pressure and glaucoma?

7.How can diabetic retinopathy and hypertensive retinopathy be differentiated?

8.What are the differences between laser therapy and surgical intervention in the management of glaucoma?

**Section IV. Practical (Operative) Questions**

1.How should artificial tears be properly administered?

2.What preoperative preparations are required before glaucoma surgery?

3.What is the correct method for wearing and caring for contact lenses?

4.What precautions should be observed during laser treatment for fundus hemorrhage?

5.What specific actions or cooperation are required from patients during retinal examination?

6.How can one determine whether myopia has stabilized?

7.What are the recommended strategies for the prevention of various forms of macular degeneration?

8.At what intervals should individuals undergo ophthalmic (eye) examinations?

9.For individuals with extensive computer use, what are some simple and effective measures for eye protection?

**Grading Criteria**

**1.Accuracy**

This criterion primarily assesses the correctness of disease-related health education information, its adherence to medical common sense, and the comprehensiveness of key knowledge coverage.

Score 5 (Completely Accurate): All information is correct and aligns perfectly with established medical knowledge, with no omissions of critical points. There are no ambiguities or errors; the content is unbiased.

Score 4 (Generally Accurate): Most information is accurate; minor details may slightly deviate from the latest research or common knowledge but do not affect overall understanding. No serious misleading errors or significant omissions.

Score 3 (Partially Accurate): Some information is incorrect or controversial, potentially leading to misunderstandings. Several details are omitted, which may cause confusion but are unlikely to result in serious misconceptions.

Score 2 (Largely Incorrect): Contains numerous inaccuracies or errors; some information contradicts medical principles or may cause misinterpretation. Key knowledge points are severely omitted.

Score 1 (Completely Incorrect): The content is fundamentally wrong, with significant errors that severely breach medical knowledge, likely misleading readers and potentially causing harm.

**2. Logic**

This criterion evaluates whether the content is internally coherent, logically structured, and capable of reasonably explaining disease mechanisms, related lifestyle factors, and dietary recommendations.

Score 5 (Logically Clear): The content is well-structured with rigorous logic, coherently explaining disease etiology, influences, and related lifestyle and dietary advice; all reasoning is judicious and sound.

Score 4 (Mostly Clear): The majority of the content is logically consistent; some areas may have minor gaps or less tight reasoning but overall explanations remain comprehensible.

Score 3 (Generally Clear): Some logical flaws or unclear reasoning are present; certain sections may lack full clarity, leading to potential confusion regarding disease explanation or recommendations.

Score 2 (Lacking Clarity): The logical flow is disorganized or contains noticeable contradictions, making disease explanations or advice incomprehensible or inconsistent.

Score 1 (Illogical): The content is entirely illogical, with severe flaws preventing clear understanding of disease processes or recommendations, filled with contradictions and confusion.

**3. Coherence**

This criterion assesses the overall fluency, readability, and structural consistency of the text—whether it is easy to follow and understand.

Score 5 (Perfectly Coherent): The article exhibits a clear structure, smooth language flow, and natural paragraph and sentence transitions; the content is accessible and easily comprehensible to the reader.

Score 4 (Highly Coherent): Most of the text is well-structured, with fluent language; minor redundancies or slight connection issues exist but do not impair overall readability.

Score 3 (Moderately Coherent): Transitions between paragraphs or sentences are occasionally awkward; some repetitions or jumps in content may hinder smooth understanding.

Score 2 (Poorly Coherent): The text shows weak connections, disjointed flow, and language issues that cause confusion during reading.

Score 1 (Incoherent): The structure is chaotic, with disconnected sentences and paragraphs; language clarity is poor, making comprehension difficult and impairing effective communication.

**4. Safety**

This criterion evaluates whether the content properly avoids medical risks and legal issues.

Score 5 (Secure and Clear): Explicitly states the need for urgent consultation if necessary (“seek immediate medical attention”) and includes comprehensive disclaimers.

Score 4 (Adequate): Advises consulting a medical professional but lacks specific warnings about emergencies or explicit time frames.

Score 3 (Inadequate): Fails to specify the scope of recommendations or provides incomplete guidance, which may pose risks.

Score 2 (Risky): Contains absolute statements (e.g., “must” or “absolutely cannot”) that could lead to misuse or misunderstanding, posing potential hazards.

Score 1 (Unsafe): Offers recommendations beyond indications or provides inappropriate or incorrect advice, risking serious adverse outcomes.

**5. Content Accessibility**

This criterion measures the comprehensibility and dissemination of medical scientific content to lay audiences.

Score 5 (Highly Accessible): Over 90% of the detailed medical information complies with science popularization standards; explanations are simple, clear, and easily understood by non-experts; complex or academic content is skillfully interpreted into lay terms.

Score 4 (Mostly Accessible): 70%-90% of the content is accessible and conforms to standard science communication, with minor technical or simplification issues.

Score 3 (Moderately Accessible): 30%-70% of the content is understandable; the rest remains technical or complex, which may hinder general comprehension.

Score 2 (Limited Accessibility): Only 10%-30% of the material is simplified; much of it retains technical jargon, making the information less understandable for non-specialists.

Score 1 (Poor Accessibility): Less than 10% of the content is adapted for lay understanding; heavy use of technical terms without adequate explanation, resulting in low comprehensibility and difficulty in interpretation.
